# Aging safely in Alentejo – understanding for action - preventing falls and violence against older people: study rationale, aims, design, and preliminary results

**DOI:** 10.1186/s12889-021-10807-8

**Published:** 2021-11-10

**Authors:** Catarina Pereira, Jorge Bravo, Gorete Reis, Felismina Mendes

**Affiliations:** 1 Departamento de Desporto e Saúde, Escola de Saúde e Desenvolvimento Humano, Universidade de Évora, Largo dos Colegiais 2, Évora, Portugal; 2 Comprehensive Health Research Centre (CHRC), Universidade de Évora, Évora, Portugal; 3 Escola Superior de Enfermagem São João de Deus, Universidade de Évora, Évora, Portugal

**Keywords:** Accidental fall, Elderly, Risk factors, Elder abuse, Prevention

## Abstract

**Background:**

Falls and violence against older people might represent a joint public health problem, as both may result in injury, fear, social isolation, sedentary behavior and dependence or even death. The ESACA project “Aging safely in Alentejo - Understanding for action” was designed to promote the healthy aging of older people in Alentejo by preventing the occurrence of falls and violence. This study aimed to report the ESACA protocol and the preliminary outcomes.

**Methods:**

The ESACA study has a twofold design as a cross-sectional study that included retrospective and prospective surveys. The participants were 508 community-dwelling older people. Assessments included falls, the risk of violence against older people, sociodemographic characteristics, health-related measurements, fear of falling, anthropometric measures and body composition, functional physical fitness, physical activity, and environmental hazards.

**Results:**

Among the participants, 43% were fallers, 21% were recurrent fallers, and 22% were victims of one or more kinds of violence (psychological: 17.1%, physical: 5.6%, and patrimonial: 3.0%). Moreover, the cumulative results suggested high risk on several risk factors for falling (7 factors: 0.6% to 2 factors: 17.4%) and of  violence (26.7%).

**Conclusions:**

In the ESACA project, a wide range of potential influencing factors on falls and violence risk factors were measured, and comprehensive quality control measures were applied. Overall, the results suggest that for falls and violence prevention strategies to be effective, it is essential to evaluate, diagnose, and inform all stakeholders in a directed and useful way. Moreover, we believe that our project outcomes may help change mindsets and behaviors by involving people in active aging and well-being programs that promote exercise and avoid isolation.

## Background

Falls are widely described as “an unexpected event in which the participants come to rest on the ground, floor, or lower level” [1], with its incidence in community-dwelling older people reported worldwide [2]. Research has pointed to an incidence of falls between 20 and 30% in people aged 65 and over and increasing for those over 70 years of age, regardless of gender or nationality, making falls and consequent injuries a major public health problem of international concern [3].

The consequences of a fall affect the quality of life of the older person. In addition to fall-related injuries, falls can result in decreased physical function and self-confidence in older people, often increasing the fear of falling, social isolation, sedentary behavior and dependence [3–8]. As a result, these restrictions may increase the risk of further falls by contributing to a deterioration in physical, cognitive, psychological and social abilities. Therefore, a consequence of falling, depending on its severity, is that direct and indirect costs of health care can also increase, compromising not only the national health systems [9] but also the informal caregivers who are faced with the need to support the people in their care, affecting the maintenance of their professional activities [10].

Despite being usually addressed separately, falls and violence against older people may represent a joint security problem. Beyond falls, violence against older people has also emerged as one of the greatest challenges for society [11]. Violence against older people has been recognized as a social and medical problem in the last few years, although this is not a recently developed issue [12, 13]. Violence against older people has been described as “the use of intentional force or power, by action or threat, against oneself or another, a group or a community causing or having the probability of causing physical or psychological damage, deprivation, death and the disruption of development” [14]. Research has played a critical role in mapping the prevalence and impact of violence against older people. A review found that overall rates of violence against older people ranged between 3.2 and 27.5% [15]. Moreover, 0.7% of older European people reported sexual abuse, 2.7% reported physical maltreatment, 3.8% reported patrimonial abuse, and 19.4% reported mental abuse in the last 12 months [16]. This phenomenon has highlighted a need for health and social care practices to identify, prevent, and intervene in cases of violence against older people [17].

The prevention of falls and violence in older people represent a current challenge for society. This challenge is accentuated in the Portuguese population living in the Alentejo region due to its demographic characteristics, with greater than 24% of its population over 65 years of age [18]. Therefore, it is essential to investigate falls and violence in older people living in Alentejo region (Portugal).

### The ESACA project

The ESACA project “Aging safely in Alentejo - Understanding for action” intended to promote the healthy aging of older people in Alentejo by preventing the occurrence of falls and violence. First, this project aimed to diagnose the incidence of falls and violence in older people living in Alentejo. Afterwards, the main risk factors and characteristics of fall occurrence and violence against older people were investigated (“understanding”). Finally, the ESACA project sought to design and implement strategies to promote healthy aging through preventive programs for fall occurrence and violence against older people (“action”). Thus, the present study aimed to report the ESACA study protocol and the preliminary outcomes.

## Study design and methods

### Design

The ESACA project has a twofold design as a cross-sectional study that included a fall retrospective survey as well as a fall prospective survey. Voluntary participant recruitment was conducted in six councils of the Alentejo region of continental Portugal - Arraiolos, Estremoz, Évora, Reguengos de Monsaraz, Viana do Alentejo, and Vidigueira.

Two main studies were carried out within the cross-sectional survey. Study one sought to screen the risk of falls in older people living independently in the community, and study two sought to screen for the risk of violence against community-dwelling older people. The retrospective survey was carried out side-by-side with the cross-sectional survey and assessed fall occurrences in the previous 12 months as well as the circumstances surrounding each fall. The prospective survey consisted of phone calls, by the rater who made the initial screening, that were performed 6 and 12 months after the first screening to record the rate of falls and the circumstances surrounding the falls.

### Participants

Samples were drawn in a similar fashion within each study location and involved compiling a list of community settings (health, recreational, sports, cultural and senior centers). Some volunteers for this study were also enrolled by means of the distribution of pamphlets and radio advertisements.

The criteria for community-dwelling participants included older people aged at least 65 with independent mobility, absence of recent injuries that have caused temporary immobilization, deafness or blindness, and absence of severe cognitive impairment in accordance with the Folstein Mini-Mental State Examination (MMSE) (i.e., scoring ≥9) [19], which would have impaired questionnaire comprehension and/or functional test completion. This study was approved by the University of Évora Ethics Committee for research in the areas of human health and well-being (reference number 16–012) and was performed in accordance with the Declaration of Helsinki. All participants provided written informed consent.

### Sample size

To estimate the minimum representative sample size, while considering the National Census [18], the web-based epidemiologic and statistical calculator for public health OpenEpi (Open Source Epidemiologic Statistics for Public Health, EUA) version 3.01 was used [20]. A sample of 385 older people ensured representativeness (90% CI). Five hundred thirteen community-dwelling older people, including 399 females (73.2 ± 5.6 years old) and 114 males (74.0 ± 6.1 years old), agreed to participate in the study and were assessed with the measures for studies one and two.

Participant recruitment started in January 2017 and ended in December 2017. The data collection lasted from April 2017 to January 2018 and was performed at the Superior Nursing School Laboratory at the University of Évora, Portugal.

### Ethical issues

Ethical approval was granted by the Universidade de Évora - Comissão de Etica para a Investigação Científica nas Areas de Saúde Humana e Bem-Estar (reference number 16–012), and written informed consent was obtained from all participants before data collection.

### Procedures

Since the sampling involved a large number of evaluations, each test was performed by the same rater throughout the data collection period, thus reducing the error associated with the measurements. The raters (who had graduated in sports sciences or nursing) received training in the procedures specialized exclusively in the application of questionnaires in the form of an interview (including cognitive, retrospective falls and violence assessment) or in the application of functional physical fitness tests and body composition, and were blind to the objectives of future studies. Regarding the data collection, each participant started the procedures with the interview, where the rater completed the questionnaires based on the verbal responses of the participant. Then, functional physical fitness tests were performed, and body composition was assessed. The evaluation process lasted approximately one and a half hours per participant. At the end of the evaluation, an individual report with the test results and rating was provided to each participant.

Follow-up evaluations were carried out by telephone call at 6 and 12 months after the first assessment by the same rater who had applied the questionnaire and involved updating the status for falls occurrence.

Data were collected from ten participants at a one-week interval between the test and retest for all the tests performed, and intra-rater reliability estimates ranged from 0.722 to 0.999 as calculated with Spearman or Pearson bivariate correlations [21].

### Common screening protocols

Assessments included falls, the risk of violence against older people, sociodemographic characteristics, health-related outcomes, fear of falling, anthropometric measures and body composition, functional physical fitness, physical activity, and environmental hazards.

#### Falls

Falls were defined as “an unexpected event in which the participants come to rest on the ground, floor, or lower level” [1]. Therefore, falls resulting from risky and dangerous circumstances or traffic accidents were not considered. Retrospective falls (in the previous 12 months) were assessed through a questionnaire completed by the rater in the form of an interview, and the circumstances surrounding each fall (such as the reason for the fall, outdoor/indoor fall, the action that was taken, and the consequences of the fall—severe injury: serious abrasion, strained muscles, torn muscles, sprains, dislocations and fractures; light injuries: slight scratches and/or edema [22]) were assessed as double-checks for false-positive answers. Prospective falls were assessed throughout telephone calls 6 and 12 months after the initial screening, and the double-checks for false-positive answers were repeated. A nonfaller was defined as a subject who had not fallen in the previous 12 months, a faller as a subject who had fallen at least once in this period, and a recurrent faller as a subject who had fallen more than once in the same period [22, 23].

#### The risk of violence

The instrument used to collect the data related to the risk of violence against older people living in the community was adapted from the Elder Abuse and Neglect-Risk Assessment Tool (E-IOA) [24] and included contributions from the Vulnerability to Abuse Screening Scale (VASS) adapted to the Brazilian reality [25]. This conjunction resulted in the Scale of Evaluation of the Risk of Violence against Non-institutionalized Older People (ARVINI), consisting of 36 questions with two response possibilities, “yes”, “no”, scored as 1, 0, respectively. No answers were also recorded. The 36 items aim to identify the risk of violence from four dimensions: social support and isolation network (1 to 12); family context (13 to 25); cognitive and emotional difficulties (26 to 30), and patrimonial issues (31 to 36), which correspond to four dimensions present in the World Health Organization definition (physical, psychological, sexual and patrimonial violence) but does not integrates the dimension of neglect [26]. The total score was obtained by summing the values of each item, with higher scores on the ARVINI scale indicating a greater risk of violence. The preliminary results of this scale were considered adequate in terms of reliability. The calculation of Cronbach’s alpha coefficient was 0.916, proving its internal consistency [27].

#### Sociodemographic characteristics

All the participants were assessed for gender, age, retirement age, education (school years), and monthly income (€).

#### Health-related outcomes

The participants listed their diagnosed chronic diseases from a total of 24 chronic diseases and reported the additional diagnosed diseases. Subsequently, the rater confirmed the information by crossing it with the answers related to the current medication, verifying the coherence between answers. The presence or absence of each chronic disease was considered as well as the total number of chronic diseases. Physical impairments, namely, frequent dizziness, foot problems, involuntary loss of urine, hearing problems, poor vision, and occasional loss of balance [28], were assessed. Health conditions variable was defined for each participant by the sum of the number of diagnosed chronic diseases and of the number of self-reported physical impairments.

Depressive symptoms were assessed through the Portuguese version [29] of the Geriatric Depression Scale 15 (GDS-15) [30]. The final score was computed by summing the scores of the 15 items, and participants were classified as without depression for scores of 5 points or less, with mild depression for scores between 6 and 10 points, and with severe depression for scores between 11 and 15 points [29].

Daytime sleepiness was measured through the Epworth Sleepiness Scale (ESS), consisting of eight items [31], where the participants were asked to rate their chances of falling asleep in eight different daily life situations on a four-point scale. The ESS total score is the sum of the item scores (recoded from 0 to 3), resulting in a scale ranging between 0 and 24 points.

Cognitive impairments were assessed using the Portuguese version of the MMSE [32], with an internal structure of 20 individual tests covering 11 domains, including orientation, registration, attention or calculation (serial sevens or spelling), recall, naming, repetition, comprehension (verbal and written), writing, and construction, for a total possible score of 30 points. The participants were categorized as having cognitive impairment or without cognitive impairment based on the cutoffs established for the Portuguese population (score 22 with 0–2 years of school, score 24 with 3–6 years of school and score 27 with ≥7 years of school) [33].

#### Fear of falling

The fear of falling was assessed by the shortened version of the Falls Efficacy Scale (FES-I) [34]. Participants were asked how concerned they felt about falling while performing each of the 16 everyday activities listed in the FES-I. Each item was rated on a 4-point scale from 1 = not at all concerned; 2 = somewhat concerned; 3 = fairly concerned; and 4 = very concerned, generating a total score from 16 to 64.

#### Anthropometric measures and body composition

Standing height (cm) was measured with a stadiometer (Seca 770, Hamburg, Germany), and weight (kg) was measured using an electronic scale (Seca Bella 840, Hamburg, Germany). Afterwards, both measures were used to compute body mass index (m/kg^2^). Body composition was measured by bioimpedance (Omron BF 511, USA) to evaluate body fat and lean mass [35].

#### Functional physical fitness

Functional fitness was assessed by using the Senior Fitness Test (SFT) and focused agility/dynamic balance, lower and upper body strength, lower and upper body flexibility and aerobic endurance, which were evaluated by the following tests: 8-ft up-and-go (s), 30-s chair stand (repetitions), arm curl (repetitions), chair sit-and-reach (cm), back scratch (cm) and 6-min walk test (m), respectively [36].

Multidimensional balance was assessed by the Fullerton Advanced Balance (FAB) Scale [21], resulting in the final score from the sum of points obtained in each of the 10 FAB tests, rated between 0 (worst) and 4 (best) with total scores ranging between 0 and 40 points.

The perception and stepping-forward boundaries were measured by the Stepping-Forward Affordance Perception Test (SF-APT), whose protocol is described in detail in a recent publication [37]. The SF-APT measurements are based on the relationship between the “estimated” stepping-forward distance (cm) and the “real” stepping-forward distance performed (cm). Posterior computation allows the generation of the following variables: algebraic-error (real performance – estimation), absolute-error (|algebraic-error|), and error-tendency which relates to error direction (overestimation: real<estimated vs. underestimation: real>estimated) [38].

Self-perceived physical function was assessed by the community-dwelling participants’ responses to the 12 items on the Composite Physical Function (CPF) Scale [39], indicating whether they could not perform the activity at all (score 0), do it with difficulty or with help (score 1) or simply could do the activity (score 2). The total CPF score could range from 0 to 24 points. The participants were categorized as moderate-high functioning (score: 18–24) or as low functioning (score < 18).

#### Physical activity

Habitual physical activity and sedentary behavior were assessed using the short version of the International Physical Activity Questionnaire (IPAQ) [40]. This questionnaire quantifies the metabolic expenditure, based on the metabolic equivalent (MET), for different activities considering the relationship between the minutes per week spent in different intensities of daily physical activity: walking (3.3 MET), moderate activity (4.0 MET) and vigorous activity (8.0 MET). Total metabolic expenditure (MET-min/week) was calculated by determining the time (min/day) and frequency (day/week) spent on each of these activities. Supervised exercise (hr/week) was also assessed by means of a questionnaire.

#### Environmental hazards

Environmental hazards were evaluated in community-dwelling participants regarding both the interior and exterior of the dwelling and also taking into account the presence of animals and habitual footwear. The presence of each listed environmental hazard was checked for each participant, and the total number of hazards was counted (minimum: 0, maximum: 34) [41].

### Statistical analysis

Descriptive analyses were performed, and data are shown as means and standard deviations or as percentages. Analyses were performed using the SPSS software package (version 24.0 for Windows, IMB Statistics). A value of *p* ≤ 0.05 was considered statistically significant for all analyses.

#### Study one

Comparisons between groups (nonfallers vs. fallers, and nonfallers vs. recurrent fallers) were performed by independent Student’s t tests for quantitative variables and chi-square tests for nominal variables after checking that the respective assumptions were met.

Low-high risk cutoffs for the risk of falling, namely, the cutoffs from which people will be classified as having a high risk of falling, were based on the literature and were as follows: GDS ≥ 5 points [42]; ESS ≥ 10 points [43]; MMSE < 24 points [44]; FES-I (fear of falling) ≥ 28 points [45]; SFT test 30-s chair stand ≤15 repetitions [46]; SFT test 6-min walk test ≤320 m [47]; SFT test 8-ft up-and-go ≥13.5 s [48]; FAB scale score ≤ 25 points [49]; SF-APT (overestimation suggests higher risk of falling) [37]; and IPAQ total physical activity < 1125 MET-min/week [22].

The percentage of participants at high and low risk of falling was calculated relative to each of these risk factors and the respective cutoff. An examination of the presence/absence of simultaneous factors indicating a high risk of falling was performed by analyzing the data distribution.

#### Study two

The occurrence of violence against the study participants and the main forms of violence were determined and categorized by age ( ≤ 80 or >  80), gender, education level (≤12 years or >  12 years), monthly income (< 550 €; 550–950 €; and ≥ 950 €), cognitive impairment (yes or no), depression (absence, mild or severe) and physical functioning (low or moderate-high) and are shown as percentages. The low-high risk cutoff for violence was defined for the ARVINI scale as 4.5 points [50]. The percentage of participants at risk of violence and not at risk was determined.

## Results

### Response rates

**Fig. 1:**
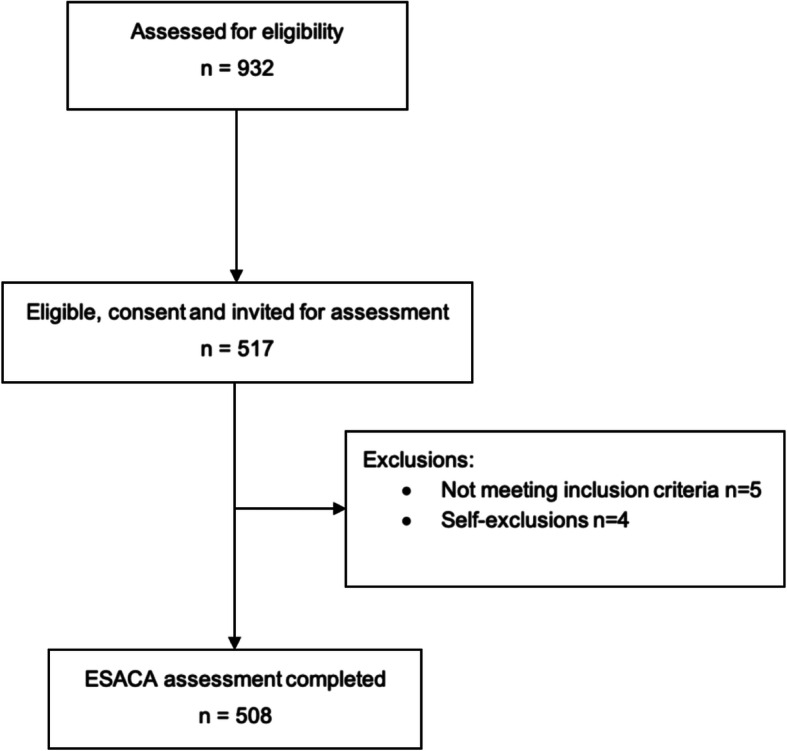
Flow diagram of ESACA study recruitment and assessment

Of the 932 older people formally invited to participate in the study (Fig. 1), 517 were eligible and consented to participate in the study, of whom 5 did not meet the inclusion criteria and 4 dropped out. Thus, 508 community-dwelling older adults actually participated in the study.

Response patterns were similar across councils/gender groups. Table 1 gives detailed response rates for individual components of testing by council and gender.

**Table 1:** Response rates, by council and gender, within elements of testing in the ESACA project

		Heath-related outcomes		Body composition		Fall-related outcomes		Functional physical fitness		Physical activity		Violence risk-related outcomes	
		n	%	n	%	n	%	n	%	n	%	n	%
Arraiolos	Men	8	100	8	100	8	100	8	100	8	100	8	100
	Women	55	98.2	55	98.2	56	100	56	100	55	98.2	55	98.2
Estremoz	Men	13	100	12	92.3	13	100	12	92.3	12	92.3	12	92.3
	Women	31	93.9	32	97	32	97	33	100	31	93.9	31	93.9
Évora	Men	74	97.4	72	94.7	75	98.7	75	98.7	74	97.4	73	96.1
	Women	199	98	200	98.5	203	100	202	99.5	203	100	203	100
Reguengos	Men	0	0	0	0	0	0	0	0	0	0	0	0
	Women	26	100	26	100	26	100	26	100	26	100	25	96.2
Viana do Alentejo	Men	12	100	12	100	12	100	12	100	12	100	12	100
	Women	34	94.4	36	100	34	94.4	36	100	34	94.4	34	94.4
Vidigueira	Men	4	80	5	100	5	100	4	80	5	100	5	100
	Women	40	100	40	100	40	100	40	100	39	97.5	39	97.5
Total	All men	111	97.4	109	95.6	113	99.1	111	97.4	111	97.4	110	96.5
	All women	385	97.7	389	98.7	391	99.2	393	99.7	388	98.5	387	98.2
	All sample	496	97.6	498	98	504	99.2	504	99.2	499	98.2	497	97.8

### Study one

Analyzing the retrospective fall incidence in community-dwelling older adults, it was observed that 43.3% of the participants were fallers, of whom 21.1% were recurrent fallers; the percentage of women who were fallers (46.9%) and recurrent fallers (23.1%) was higher than the percentage of men (31.6 and 14.1%, respectively), *p* < 0.05. In addition, 60.1% of the fallers and 69.9% of the recurrent fallers experienced fall-related injuries; that is, 25.7% of the community-dwelling participants had been injured. In fact, retrospective falls totaled 508, of which 26.1% resulted in injury: 392 light injuries (light scratches or edema) and 72 severe injuries (serious abrasion, strained muscles, torn muscles, sprains, dislocations and fractures).

As can be observed in Table 2, fallers were less healthy than nonfallers (i.e., had ~ 1 more health condition, ~ 1 more geriatric depression score point, ~ 1 less on cognitive status); had a poorer body composition (i.e., ~ 3% more fat body mass); were less fit (performed 1 fewer repetition on lower and upper strength tests; went ~ 34 m less distance in the aerobic endurance test; scored ~ 3 points lower on the multidimensional balance test; and took ~ 1 s longer on the mobility test); were less independent with activities of daily living (scored 2 points lower on the physical function scale); and were more afraid of falling (scored ~ 4 points more on the fear of falling scale), *p* < 0.05. There were differences that were enhanced when recurrent fallers and nonfallers were compared: recurrent fallers had ~ 2 more health conditions, ~ 2 more points on the geriatric depression scale score, ~ 2 points lower on the cognitive measure, ~ 3% more fat body mass; they performed 1 fewer repetition on lower and upper strength tests, went ~ 46 m less distance in the aerobic endurance test, and scored ~ 4 points lower on the multidimensional balance test; they took ~ 1 s longer on the mobility test; and they scored ~ 2 points lower on the physical function scale and ~ 6 points higher on the fear of falling scale, *p* < 0.05.

**Table 2:** Participants characteristics as regards risk factors for falling according to retrospective falls occurrence

Variable	Nonfallers	Fallers	Recurrent fallers	Low-high risk for falling cutoff	Low risk prevalence	High risk prevalence
Age (yrs)	73.2 ± 6.1	73.6 ± 6.9	73.3 ± 7.5	–	–	–
Gender						
Female	53.1%	46.9%	23.1%	–	–	–
Male	68.4%	31.6%	14.1%			
Education (yrs)	5.3 ± 4.0	5.1 ± 3.7	4.8 ± 3.5	–	–	–
Health conditions (n)	5.4 ± 2.8	6.9 ± 3.2 ^a^	7.5 ± 3.2 ^b^	–	–	–
Geriatric depression score (0–15)	3.5 ± 3.0	4.8 ± 3.5 ^a^	5.5 ± 3.8 ^b^	≥ 5	91.6%	8.4%
Daytime sleepiness score (0–24)	4.5 ± 3.7	4.3 ± 3.7	4.0 ± 3.7	≥ 10	91.9%	8.1%
Cognitive status score (0–30 points)	26.6 ± 4.1	25.5 ± 5.4 ^a^	24.5 ± 6.6 ^b^	< 24	83%	17%
Fear of falling score (16–64 points)	23.1 ± 6.5	27.1 ± 8.6 ^a^	29.2 ± 9.8 ^b^	≥ 28	72.4%	27.6%
Body mass index (kg/m^2^)	28.5 ± 4.0	29.1 ± 4.2	29.3 ± 4.1	–	–	–
Fat body mass (%)	30.7 ± 7.9	33.7 ± 6.9 ^a^	34.0 ± 6.7 ^b^	–	–	–
Lower body strength (rep)	13.2 ± 4.6	11.9 ± 4.7 ^a^	11.8 ± 4.1 ^b^	≤ 15	77.2%	22.8%
Upper body strength (rep)	15.2 ± 4.8	14.4 ± 5.2 ^a^	13.9 ± 5.0 ^b^	–	–	–
Lower body flexibility (cm)	−2.7 ± 10.3	−3.8 ± 10.8	−3.1 ± 10.0	–	–	–
Upper body flexibility (cm)	−13.6 ± 12.0	−14.8 ± 12.7	−15.1 ± 12.6	–	–	–
Aerobic endurance (m)	447.3 ± 99.5	413.1 ± 111.7 ^a^	401.7 ± 105.1 ^b^	< 320	84.9%	15.1%
Agility/dynamic balance (sec)	6.8 ± 2.4	7.5 ± 2.6 ^a^	7.9 ± 2.8 ^b^	≥ 13.5	96.9%	3.1%
Multidimensional balance (0–40 points)	30.7 ± 6.5	27.8 ± 7.4 ^a^	27.1 ± 8.1 ^b^	≤ 25	78.7%	21.3%
Stepping-forward error-tendency						
Underestimation	81.0%	73.0%	74.8%	–	–	–
Overestimation	19.0%	27.%	25.2%			
Physical functioning score (0–24 points)	21.3 ± 3.7	19.3 ± 4.9 ^a^	19.1 ± 5.1 ^b^	–	–	–
Physical activity (MET-min/wk)	1818.4 ± 2464.2	1957.0 ± 2669.2	1938.9 ± 2523.4	< 1125	60.6%	39.4%
Environmental hazards number (0–34)	14.4 ± 3.4	14.1 ± 3.6	13.8 ± 3.5	–	–	–

Data are mean ± standard deviation or percentage in case of prevalence analysis. ^a^ Significant difference between non-faller and fallers. ^b^ Significant difference between non-faller and recurrent fallers

The data analysis exposed in Table 2 also showed that there were many participants at high risk of falling according to the low-high risk for falling cutoffs established for the study variables. Depending on the variable, the percentage of participants at high risk of falling varied from 3.1% on agility/dynamic balance (spent more than 13.5 s on up and go test) to 39.9% on total physical activity (spent < 1125 MET-min/wk. on physical activity). In addition, the data distribution analysis showed that: a) there were participants with multiple risk factors for falling showing results of high risk (7 factors: 0.6%; 6 factors: 0.7%; 5 factors: 2.8%; 4 factors: 7.4%; 3 factors: 9.3%; 2 factors: 17.4%); b) 32.8% of the participants were at high risk in one factor; and c) only 14.3% of the participants were not at high risk of falling across all factors.

Finally, the perspective falls data analysis showed that 1 year after the cross-sectional survey, the percentage of fallers decreased to 35.5%. Nonetheless the percentage of fallers who experienced a fall-related injury (67.5%) did not decrease, the total percentage of participants experiencing an injury decreased to 23.9%, of which 44.3% corresponded to light injuries and 55.7% corresponded to severe injuries.

### Study two

Data analysis showed that 21.6% of the studied participants were victims of some form of violence and that the most frequent form of violence was psychological (17.1%), followed by physical violence (5.6%) and patrimonial violence (3.0%). Sexual violence did not show a significant expression in the studied sample. It should be highlighted that while the percentage of men who were victims of violence was 16.5%, the percentage of women was 23.1%. As shown in Table 3, which presents several main risk factors for violence reported in the literature and identified in the present study, women were the preferred victims of violence, either psychological, physical or patrimonial, with prevalence rates of 18.3, 6.7, and 3.1%, respectively. From another perspective, the poorly educated older people, with ≤12 years of school, suffered more violence in different forms (psychological: 10.4%, physical: 3.4% and patrimonial: 3.8%), and the older people with lower incomes (< 550€/month) were most often victims of violence (psychological: 9.4%, physical: 3.8% and patrimonial: 3.4%). Regarding cognitive status, people with cognitive impairment were more susceptible to being victims of psychological (17.3%), physical (6.3%) and patrimonial (3.2%) violence than people without cognitive impairment (16.1, 0 and 1.8%, respectively). Additionally, persons with severe depression were more prone than those without severe depression to be victims of violence (psychological: 37.0%, physical: 22.2% and patrimonial: 7.4%).

**Table 3:** Risk factors for the main forms of violence against older adults and respective prevalence

Risk Factors		Psychological violence (%)	Physical violence (%)	Patrimonial violence (%)
Age	≤ 80 years	18.4	6.7	3.1
	> 80 years	10.4	0	2.6
Gender	Female	18.3	6.7	3.1
	Male	12.8	1.8	2.8
Education	≤ 12 years	10.4	3.4	3.8
	> 12 years	1.8	0.6	0.4
Monthly income	< 550 €	9.4	3.8	3.4
	550–950 €	4.0	1.0	2.0
	> 950 €	2.6	0.8	1.2
Cognitive impairment	No	16.1	0	1.8
	Yes	17.3	6.3	3.2
Depression	Absence	11.9	3.9	2.5
	Mild	26.3	7.0	3.5
	Severe	37.0	22.2	7.4
Physical functioning	Low	17.3	6.5	3.9
	Moderated-high	15.2	5.5	2.7

Finally, older people with low physical functioning were also more prone to be victims of violence (psychological: 17.3%, physical: 6.5% and patrimonial: 3.9%) than those with moderate-high functioning (psychological: 15.2%, physical: 5.5% and patrimonial: 2.7%). With some surprise, it was observed that, in contrast with the younger old adults (< 80 years), the older old adults (≥ 80 years) less frequently reported being victims of violence (psychological: 10.4%, physical: 0% and patrimonial: 2.6%).

Considering the violence cutoffs regarding the ARVINI scale (score 4.5), 26.7% of the older people were at risk of violence.

## Discussion

The present study aimed to report the ESACA study protocol and describes the measures and the preliminary outcomes regarding the risk of falls and violence. This main inference from this study was that both the incidence of falls and the number of older persons who are victims of violence are too high. Our cross-sectional and retrospective survey showed that almost half of the participants fell at least once in the previous 12 months and that almost a quarter of these older persons were victims of violence, including physical violence, monetary violence and mainly psychological violence. These values, which in themselves are frightening, surpass the international trends concerning fall occurrences (approximately one-third of older adults fall once a year) and match the worst world scenarios concerning violence against older people (overall rates of violence against older people of 27.5%) [3, 15]. Surprisingly, it was observed that there were participants at high risk of falling who were unaware of this fact and that there were participants who were victims of violence, particularly psychological violence, who did not “know” that they were being subjected to violence. The first observation is in accordance with previous studies reporting that many older persons are aware of the consequences of falling but have a poor awareness of their own risk of falling [51]. Nevertheless, the second observation was unexpected because the literature usually reports that the institutional and governmental agents’ lack of knowledge is a barrier to violence prevention [14]; but, in this study, it became evident that the victims of violence themselves were not aware of their condition.

In the present study, the results showed that the percentages of older people who showed values above the low-risk cutoff both for the risk factors related to falls and for the risk factors related to violence were very high. Additionally, many persons had high risk based on multiple factors related to falls and violence. This observation suggests that if generalized and effective community strategies are not defined to prevent falls and violence, these problems will likely perpetuate or even worsen, as Portuguese demographic projections from 2015 to 2018 predict a decrease in the active population above 65 years from 6.7 to 3.8 million and an increase in older people from 2.1 to 2.8 million [52].

Another extrapolation resulting from data analysis was the realization that the risk factors for falls and violence were somewhat similar, with an emphasis on the female gender and social, physical, cognitive or emotional prefrailty indicators (e.g., low monthly income, high comorbidity, low fitness, low functioning, compromised cognitive performance, geriatric depression). These new findings were made possible by simultaneously approaching the two phenomena and complementing the observation of others reporting frailty as a vulnerable state associated with an increased susceptibility for multiple adverse health outcomes [53]. Although the above risk factor examples may lead us to identifying the characteristics of older people who are fallers or victims of violence, it is essential to also consider falls and violence opportunism, as these events only happen when the person is exposed to circumstances that can lead to a fall [54] or to an aggressor with the respective profile particularities [3].

Additionally, it should be noted that in the present study, a notable decrease in the prevalence of falls was observed in the studied sample from the falls retrospective survey (focusing on fall occurrences in the year “before” each participant received the individual risk report describing the risk factors contributing to fall risk) to the falls prospective survey (focusing on fall occurrences in the year “after” receiving the report). This suggests that the informed awareness of the risk of falling combined with the knowledge of which factors are present and contributing to this risk can by itself be an important and effective fall prevention measure. This finding strengthens the importance of providing the recommended risk of falling assessment for fall prevention [55].

The above findings suggest the importance of defining holistic prevention strategies to promote safe and active aging without falling and without violence. These strategies include 1) evaluation, 2) dissemination of evaluation results to all stakeholders, and 3) interventions focused on behavior change and privileging integration in community health promotion programs, including exercise practice. This last strategy may be of particular importance because community programs including exercise favor the building of community knowledge and networking to combat isolation, in addition to contributing to improving fitness and limiting the development and progression of chronic disease and disabling conditions, which make older people more susceptible to falls and violence [56, 57]. In addition to concrete and operational social concerns, such as minimizing the consequences of a low monthly income, the programs should induce improvements in the identified physical, cognitive and emotional risk factors for falls and violence associated with prefrailty (such as strength, balance, aerobic endurance, cognitive capacity and depressive states). They should include measures to promote changing stereotypes that persist in today’s society against older people and especially against women, who are the main physical, monetary and psychological violence victims reported in the present and other studies [3, 58]. Stereotypes that favor violence in the context of ageism stands out, as these are translated into social devaluation of and discrimination against people who are deprived of social role because they may be less able and/or may be dependent on third parties [50].

The major strength of this study is the large sample size of 508 participants who are representative of the older population residing in the community in Alentejo, which gives our study high external validity with a high level of statistical power. Similarly, the measurement of a comprehensive range of factors that potentially influence falls and violence risk enabled an informed judgment of important factors contributing to these events and how they vary either in the population or between subgroups. The wide range of measurements facilitated investigation of a broad range of potential confounders in the within-individual analyses. Likewise, we consider that the application of field tests, without the need for elaborate laboratory protocols, allows wide-scale applicability and enhances the ability to conduct more extensive data collections for epidemiological research.

Some limitations of the current approach can also be summarized. Despite rigorous intra-rater reliability checks made in controlled situations, differential application of study protocols in the field cannot be discounted. Likewise, the evaluation time per participant proved to be excessive; even so, comprehensive assessments are needed to develop risk prediction models, allowing the identification of the most valuable data and consequently shortening the protocol.

## Conclusion

In conclusion, in the ESACA project, a wide range of potential influencing factors on falls and violence risk factors were measured, and comprehensive quality control measures were applied. The present study results suggest that for falls and violence prevention strategies to be effective, it is essential to evaluate, diagnose, and inform in a directed and useful way all stakeholders about the evaluation results and respective interpretation, to involve older people in community programs combating isolation and privileging exercise, and to change all stakeholders’ mindsets and behavior, that is, understanding for action. The ESACA project is well placed to provide further insights into key critical questions regarding the determinants of falls and violence against older people and to what extent risk factors are prevalent.

## Data Availability

The datasets used and/or analyzed for the current study are available from the corresponding author upon reasonable request.

## Abbreviations

ESACA: Aging safely in Alentejo – Understanding for action
MMSE: Mini-Mental State Examination
E-IOA: Elder Abuse and Neglect-Risk Assessment Tool
VASS: Vulnerability to Abuse Screening Scale
ARVINI: Scale of Evaluation of the Risk of Violence against Non-institutionalized Older People
GDS-15: Geriatric Depression Scale 15
ESS: Epworth Sleepiness Scale
FES-I: Falls Efficacy Scale
SFT: The Senior Fitness Test
FAB: Fullerton Advanced Balance
SF-APT: Stepping-Forward Affordance Perception Test
CPF: Composite Physical Function
IPAQ: International Physical Activity Questionnaire
MET: Metabolic equivalent of task
SPSS: Statistical Package for the Social Sciences
IBM: International Business Machines Corporation.
